# Vaccination, and Re-Inoculation with Variolous Matter, at the Foundling Hospital

**Published:** 1805-12

**Authors:** C. Stanger

**Affiliations:** Lamb's Conduit Place


### Vaccination, and Re-inoculation with Variolous Matter, at the Foundling Hospital.

*To the Editors of the Medical and Physical Journal.*

Gentlemen,

I Presume that an account of the trials which vaccination has undergone, in the Foundling Hospital, as a preservative against small-pox, will not be deemed undeserving a place in your useful publication.

After the vast and satisfactory mass of evidence which has been accumulated, from every quarter of the globe, to establish the safety and certainty with which vaccination counteracts small-pox, the few cases here mentioned would probably not have been published, had not some late unfavourable statements excited considerable alarm for the safety of those who have undergone vaccination; and deterred many from having recourse to this preservative. The importance and estimation of the public charity in which the proofs, here recited, of the efficacy of vaccination were obtained, may also give them more weight, in the estimation of the community, than those derived from a much greater number of cases in private practice.

Inoculation with cow-pox matter was first introduced into the hospital on March 30, 1801, when two girls were vaccinated by Mr. Ramsden, surgeon to the charity, with matter supplied by Mr. Griffith, surgeon, of Lower Grosvenor Street. On April 10, two girls and six boys were inoculated with matter taken from one of the children first vaccinated. On September 15, two more girls were inoculated with vaccine fluid, a second time supplied by Mr. Griffith,

Griffith; and from those two girls and from one another, in succession, eight more children were vaccinated, during the ensuing months of the same year. One of the above children was inoculated four times before she had the disorder; but all the rest were affected from the first insertion of the vaccine fluid. The only symptoms which occurred were the usual inflammation, vesication, and scabbing of the punctured part, and, in some cases, slight febrile indisposition, which was not in any instance so considerable as to require medical assistance.

On March 24, 1802, one girl was vaccinated without being infected, and was re-inoculated on May 17, when two more girls and five boys underwent inoculation, with vaccine matter taken from a patient sent from the Small-pox Hospital. On May 25, six girls and one boy were vaccinated from the preceding patients, and excepting that the girl first inoculated had matter inserted into the arm four times, and another thrice before she underwent the disorder, neither any local or constitutional symptoms occurred in any of the children vaccinated in this year, except those which characterise the disease.

In the course of the year 1803, thirty-three children were vaccinated with matter procured by Mr. Ramsden, for the first cases, and afterwards produced by patients in the hospital. They all took the infection from the first insertion, and passed through the disorder in the same favourable manner as those vaccinated in the two preceding years, as did also thirty-four children vaccinated in the month of April in the present year.

On August 9, 1802, all the children who had been previously vaccinated, amounting to thirty-five, were inoculated with variolous matter taken from a child brought from the Small-pox Hospital to the Foundling Hospital. In most of these cases, the puncture presently healed; in some slight inflammation was produced, and in three or four cases there appeared a small acuminated pustule, which, after some days, was succeeded by a slight scab, no constitutional disorder having intervened.

In November 1804, in consequence of apprehensions excited in some of the Governors of the Foundling Hospital that vaccination might not be a permanent security against small-pox, twenty-one of the children who had been first vaccinated in 1801, and afterwards variolated in 1802, were a second time inoculated with matter taken from a child labouring under natural small-pox, and brought as before from the Small-pox Hospital.

The

The result of this trial, made three years and a half after vaccination, confirmed its preventive power. The only effects produced were slight inflammation about the puncture, in some cases; and in a few others, a small local pustule which soon disappeared. These cases afford decisive evidence, as far as their number extends, that the preservative influence of vaccination is not diminished in the above mentioned period, exceeding the term in which the eruptions and other symptoms of small-pox occurred in the patients, previously vaccinated, whose cases have been published by Mr. Goldson.

The same twenty-one patients also supply the most satisfactory evidence to invalidate Mr. Goldson's supposition, that eruptive diseases may remove the security derived from cow-pox. During the period which intervened between their being vaccinated in March 1801, and variolated in November 1804, six of them had scarlet fever and four measles. Ten of them had hooping cough also, which proves that these contagious febrile disorders, which so powerfully affect the constitution, have no influence in diminishing the efficacy of vaccination. As the first inoculation with variolous matter, in 1802, produced no constitutional affection, it cannot supply an argument against the durability of the antidote for three years and a half.

I shall feel great satisfaction if the above evidence, deriving weight from the public institution in which it was obtained, should relieve the fears of any of those who have confided in vaccination, or encourage others to have recourse to it.

I cannot omit the present opportunity of adding my tribute of admiration of Dr. Jenner, who first established the power of vaccine inoculation in preserving mankind from that loathsome and fatal disease small-pox. Whether we consider the importance of the discovery, the penetration with which he investigated, the ability with which he confirmed, or the candour with which he made it public, Dr. Jenner is justly entitled to the gratitude and admiration of mankind.

I am, &c.

C. STANGER.

*Lamb's Conduit Place, Oct. 20, 1805.*

